# Effect of Molecular Composition of Head Group and Temperature on Micellar Properties of Ionic Surfactants with C12 Alkyl Chain

**DOI:** 10.3390/molecules24030651

**Published:** 2019-02-12

**Authors:** Jarmila Oremusová, Zuzana Vitková, Anton Vitko, Marián Tárník, Eva Miklovičová, Oľga Ivánková, Ján Murgaš, Daniel Krchňák

**Affiliations:** 1 Department of Physical Chemistry of Drugs, Faculty of Pharmacy, Comenius University, Odbojárov 10, 832 32 Bratislava, Slovak Republic; 2 Department of Galenic Pharmacy, Faculty of Pharmacy, Comenius University, 832 32 Bratislava, Slovak Republic; zuzana.vitkova@stuba.sk (Z.V.); krchnak6@uniba.sk (D.K.); 3 Institute of Robotics and Cybernetics, Faculty of Electrical Engineering and Information Technology, Slovak University of Technology, 812 19 Bratislava, Slovak Republic; anton.vitko@stuba.sk (A.V.); marian.tarnik@stuba.sk (M.T.); eva.miklovicova@stuba.sk (E.M.); jan.murgas@stuba.sk (J.M.); 4 Department of Structural Mechanics, Faculty of Civil Engineering, Slovak University of Technology, 810 05 Bratislava, Slovak Republic; olga.ivankova@stuba.sk

**Keywords:** ionic surfactants, dodecyl hydrophobic part, conductivity, critical micellar concentration, thermodynamic parameters of micellization

## Abstract

The paper analyses influences of the temperature and hydrophilic groups on micellar properties of ionic surfactants with 12-carbonic hydrophobic chains. The aim is to assess the impact of hydrophilic groups and temperature on thermodynamic parameters and micellization. This knowledge is indispensable for the formulation of new dosage forms. The method uses conductometric measurements. The following hydrophilic groups are analyzed: trimethylammonium bromide, trimethylammonium chloride, ethyldimethylammonium bromide, didodecyldimethylammonium bromide, pyridinium chloride, benzyldimethyl-ammonium chloride, methylephedrinium bromide, cis and trans-[(2-benzyloxy)-cyclohexyl-methyl]-N, N-dimethylammonium bromide, sodium sulphate and lithium sulphate. Except for a few cases, there is a good agreement between values of critical micellar concentrations (CMC) and critical vesicle concentration (CVC) obtained here and those which were obtained by other authors and/or by other physicochemical methods. Values of the CMC are compared with respect to the molar masses of hydrophilic groups. It was found that CMC values increased non-linearly with increasing system temperature. The degrees of counterion binding and thermodynamic parameters, like the standard molar Gibbs energy, enthalpy and entropy of micellization are determined and discussed in detail. The results obtained will be incorporated into in silico processes of modeling and design of optimal dosage forms, a current interdisciplinary research focus of the team.

## 1. Introduction

Surfactants are amphiphilic molecules having separate lyophilic or solvophilic (solvent-loving) and lyophobic or solvophobic (solvent-hating) groups. Cationic surfactants create a large group of chemical substances consisting of derivatives of quaternary ammonium, phosphonium, iodonium, sulphonium biquanides amines etc.

Aggregation of surfactants is widely studied for various industrial and research intentions [1]. Surfactants are used in numerous applications of the fundamental and applied sciences. They serve as solubilizers, emulsifiers, detergents [2,3] and models of several biochemical and pharmacological systems.

Quaternary ammonium salts (QAS) are kinds of cationic surfactants with good water solubility in the concentrations used. Owing to their properties (low toxicity, strong surface activity, disinfectant and bactericidal properties, low price and simple preparations) they are also used in household chemicals and the chemical industry [2,4,5,6,7].

The amphiphile molecules in solution of a certain concentration have a propensity to form aggregates: micelles, vesicles, bilayers and a lot of nanostructures in diverse media [8]. Parameters of micellization, like critical micelle concentration (CMC), critical vesicle concentration (CVC), thermodynamic values, aggregate number, stableility etc. play important roles in different applications. Their properties depend on both inner and outer influences.

Inner influences are the surfactant’s structure, the length of the alkyl chain, the type of the ionic group, and the character of the counterion [6]. The association of ionic surfactants in aqueous solutions is induced by hydrophobic interactions between alkyl chains of molecules, balanced by hydration and/or electrostatic interactions [7].

Outer influences are the temperature and diverse additives: co-solvents, co-surfactants, inorganic and organic substances, organic and inorganic solutes and drugs [9,10,11]. Micelles can solubilize badly soluble substances in their hydrophobic interiors [11].

The estimate of a shape (geometry) of a surfactant aggregate is generally expressed in the packing parameter (*p*) [12]:

(1) $$P=\frac{V_{0}}{l_{C}a_{0}}$$

*V*_0_ is the volume engaged by the alkyl chain portion; *l*_C_ is the length of the alkyl chain; *a*_0_ is the hydrophilic group area

If *p* lies in the interval 0–0.333; the expected aggregates structures are spherical or ellipsoidal micelles. Single chain surfactants with bulky hydrophilic groups typically belong to this category. For *p* being in the range 0.333–0.5, the micelles are formed in the cylindrical or rod shapes. For this category, a single chain and a small hydrophilic group of surfactant is typical. For *p* being in the range 0.5–1.0 the obtained structure takes a shape of vesicles or flexible bilayer [8]. These structures are obtained from double-chain surfactants.

The CMC’s temperature dependences may, in accordance with some references, take the shape of the capital letter U [13,14]. This dependence can be used as a determinant of thermodynamic parameters of micellization—the standard molar Gibbs energy, the enthalpy, entropy and the molar heat capacity of micellization [15,16].

Applications of surfactants require detailed understanding of their physicochemical properties, for instance of the surface tension, conductivity, density, viscosity, geometry, size, CMC, degree of counter ion binding etc. This fact was an incentive for the authors to analyze effects of the temperature and types of hydrophilic group of ionic surfactants with C12–dodecyl-hydrophobic groups on values of CMC and to incorporate these effects into in silico models. The analysis uses conductometric measurements which seem to be very suitable just for the ionic surfactants. Below CMC, the conductivity of the surfactant solution is caused by the presence of free surfactant ions and counter ions [17].

Various theoretical approaches are applicable for obtaining micellization parameters, like CMC, degree of counterion binding (β) and thermodynamic parameters of micellization. Here we thoroughly discuss the standard molar Gibbs energy of micellization (Δ_m_*G*^0^), standard molar enthalpy of micellization (Δ_m_*H*^0^) and standard molar entropy of micellization (Δ_m_*S*^0^). The obtained parameters are clearly arranged into tables and compared with those acquired by some other authors, and/or by other methods [18,19,20,21,22,23,24,25,26,27,28,29,30,31,32,33,34,35,36,37,38,39,40,41,42,43,44,45,46,47,48,49,50,51,52,53,54,55,56,57,58,59,60]. We did this because exact knowledge of parameters is inevitable for synthesis of the reliable in silico means of predicting bioavailability and the effects of the final drugs.

As to the temperature, the character of head groups and the length of alkyl chain significantly influence numerous physicochemical parameters of ionic surfactants, including CMC. Our research has been aimed at obtaining accurate and precise parameters of micellization together with the related dependences. Our main research consists in the design of predictive in silico models which are to be able to predict bioavailability or even the therapeutic effect of newly synthetized/modified drugs and corresponding dosage forms. To do this, the designer must be (among other things) familiar with the thermodynamic dependences and conditions under which the drug is encapsulated into micelles. To this end, the identified parameters have been clearly arranged into tables and compared with those obtained by some other authors and/or by other methods. Moreover, two cation surfactants, namely DBDMABr cis, trans (see Table 1), were synthetized at our workplace. Identification of the related parameters is underway. So far, they have not been published and their comparison with other authors is still missing. The results obtained so far have been partially incorporated into in silico models which we synthetized with the aim of designing an optimal dosage form. That is a topic of our current interdisciplinary research.

## 2. Experimental Parts

### 2.1. Material and Equipment

**Figure 1:**
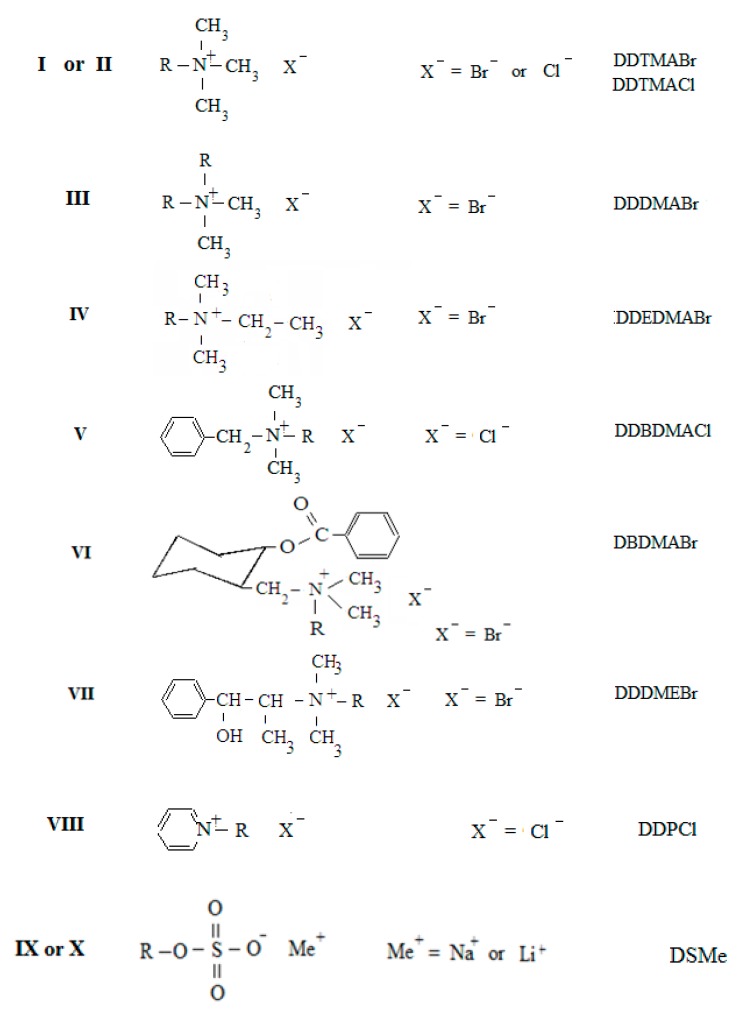
Structural formula of studied compounds. R is the dodecyl chain [CH_3_-(CH_2_)_11_-].

Eight cationic surfactants from the group of quaternary ammonium salts and two anionic surfactants from the group of alkyl sulphates were studied (Figure 1).

Fundamental characteristics of compounds—the marking of compounds, molar masses of compounds (*M_c_*), molar masses of hydrophilic part of compounds (*M*_hg_) without of the molar mass of the counter ions and the hydrophobic part of the surfactants, and melting points (m.p.)—are given in Table 1.

The surfactants were used without further purification and purity declared by supplier was 99% or better. The surfactants studied are highly soluble in water in the range of concentration and temperature investigated.

### 2.2. Method

Conductivities of solutions were measured by conductometric titration (i.e., by dilution of a more concentrated solution with redistilled water) at the temperature range 20–50 °C in a thermostatic glass cell with platinum electrodes Tetra Con 325 (cell constant K = 0.474 the cm^−1^). The device was calibrated by measuring the conductivity of solutions of potassium chloride (Merck) of different concentrations (0.001, 0.01 and 0.1 mol dm^−3^). For the measurements, we used a precise (±0.01 μS cm^−1^) digital conductivity meter Ino Lab (Swiss) was used. The electrode was inserted into a double-walled glass flask filled with the solution. Solutions were continually stirred and thermostated by the thermostat JULABO 5E (Swiss) with a precision of ±0.1 °C. The CMC values were estimated from the dependences conductivity vs. molarity [κ = *f*(*c*_surf_)], which were obtained from the measurements.

The surfactant solutions (volume 25 mL) were prepared in a wider concentration range around of the CMC values. Solutions of lower concentrations were prepared by gradual dilution by adding re-distilled water. Conductivity of the diluted solution was measured after 5 min after their preparation. The conductivity curves were measured three times for every temperature.

## 3. Results and Discussion

### 3.1. Determination of Critical Micelle Concentration (CMC)

**Figure 2:**
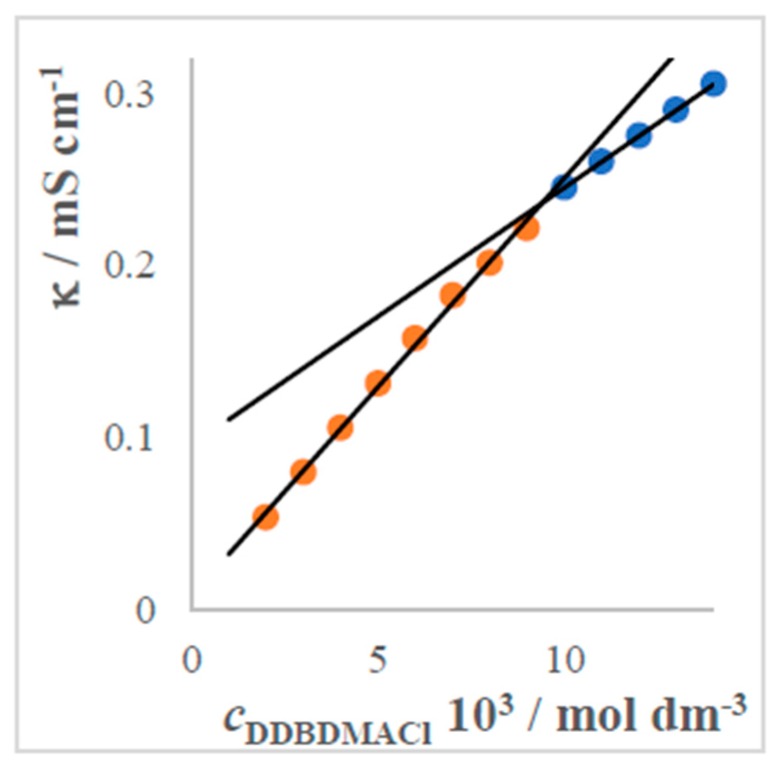
Conductivity versus molarity curves for binary system DDBDMACl (V)–water at temperature 45 °C.

The CMC is a useful parameter used in establelishing quantitative relations between the surfactant structure, physicochemical parameters, and biological activity [61]. Representative plots of aqueous solutions are presented as conductivity versus molarity dependences (Figure 2). Dependences κ = f(*c*) for temperature interval 20–50 °C exhibit very small differences of conductivity, and therefore we show only one (Figure 2). For higher concentrations of the surfactant V we obtained the second value of CMC, namely CMC_2_, as shown on Figure 2 right. The corresponding values of CMC_2_ are in Table 2.

The curves exhibit typical behaviours of surfactants. They consist of two linear parts, the intersection of which defines the CMC.

Conductivities of all systems increase with increasing concentration of the studied surfactants in the solutions. Conductivity increases due to increasing thermal energy of the molecular entities [15]. Critical micellar concentrations were calculated from the dependences’ conductivity vs. molarity (Figure 2). The establelished CMCs are summarized in Table 2.

It is generally accepted that the CMC strongly decreases with increasing alkyl chain length of the surfactant. According Lindman [62], after the addition of one (-CH_2_-) group to the alkyl chain the CMC decreases two times for ionic surfactants and three times for non-ionics.

It is possible to compute the packing parameter *p* (Equation (1)) and predict the kind of the aggregate. All studied surfactants, except for III have only one hydrophobic dodecyl chain. We have calculated the value of *p* only for single and double-long hydrocarbon chains in the surfactant´s molecule.

The quantities in Equation (1), namely *l*_C_ -alkyl chain length and *V*_0_-volume engaged by the alkyl chain portion were estimated from the following equations (suggested by Tanford [7]):

*l*_C_ = 1.5 + 1.265*n* [Å] (2)

(3) $$V_{0}=27.4+26.9n;\;[{\text{Å}}^{3}]\;\mathrm{for}\;\mathrm{single}-\mathrm{long}\;\mathrm{hydrocarbon}\;\mathrm{chain}$$

(4) $$V_{0}=54.3+27.05n;\;[{\text{Å}}^{3}]\;\mathrm{for}\;\mathrm{double}-\mathrm{long}\;\mathrm{hydrocarbon}\;\mathrm{chains}$$

*n* is a number of carbon atoms in the alkyl chain.

The hydrophilic group area—*a*_0_, the radius of a micelle—*r* and the aggregation number *N*_Agg_ were estimated by the equation (suggested by [7]):

(5) r=1.6+1.265 (n+1)+0.421m [Å]

*m* is a number of carbon atoms in substituted alkyl chain;

(6) $$N_{Agg}=\frac{\frac{4}{3}\pi_{l_{C}^{3}}}{V_{0}}$$

(7) $$a_{0}=\frac{3V_{0}}{r}\;[{\text{Å}}^{2}]$$

The calculated values of *l*_C_, *V*_0_, *a*_0_, *r*, *N*_agg_ and *p* are summarized in Table 3.

**Figure 3:**
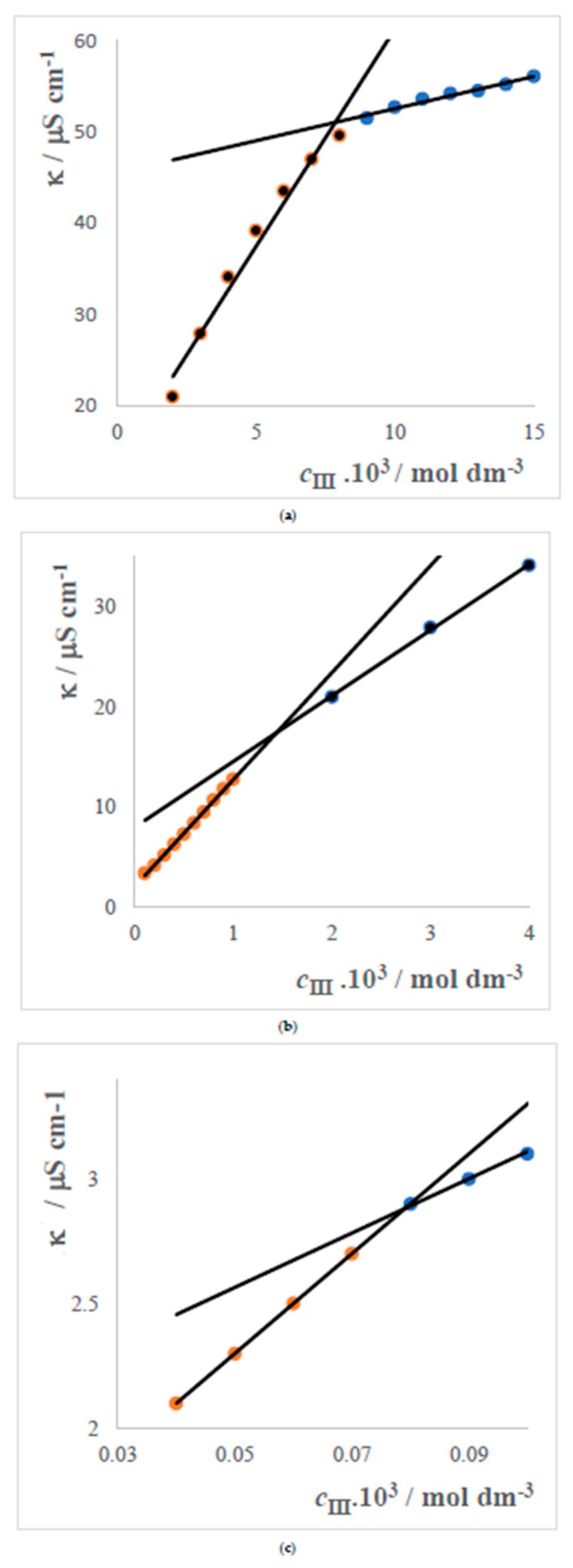
Conductivity curves of DDDMABr with values of CVCs at 25 °C. (**a**) CVC_1_ = 7.96 × 10^−3^ mol dm^−3^; (**b**) CVC_2_ = 1.66 × 10^−3^ mol dm^−3^; (**c**) CVC_3_ = 0.079 × 10^−3^ mol dm^−3^.

The results obtained for the packing parameter show that the surfactants with single long alkyl chain create spherical micelles, while didodecyldimetylamonium bromide creates vesicles. For this reason, it was decided to measure conductivity curves for the concentrations of the DDDMABr within the range (15–0.04) × 10^−3^ mol dm^−3^. For the same reason, the conductivity curve has three breaks, which are depicted in the three separate graphs. The breaks correspond to the critical vesicular concentration (CVC) as shown in Figure 3.

Similar results were obtained in [63,64,65,66,67] and they are summarized in Table 4.

An increase of CMC for increasing temperature is influenced by two opposite phenomena [16].

- The increased temperature may decrease hydration of hydrophobic groups of surfactants, which in turn supports the formation of micelles.
- The temperature accelerates breaking of the water structure around the hydrophobic group, which in turn suppresses the formation of micelles.

**Figure 4:**
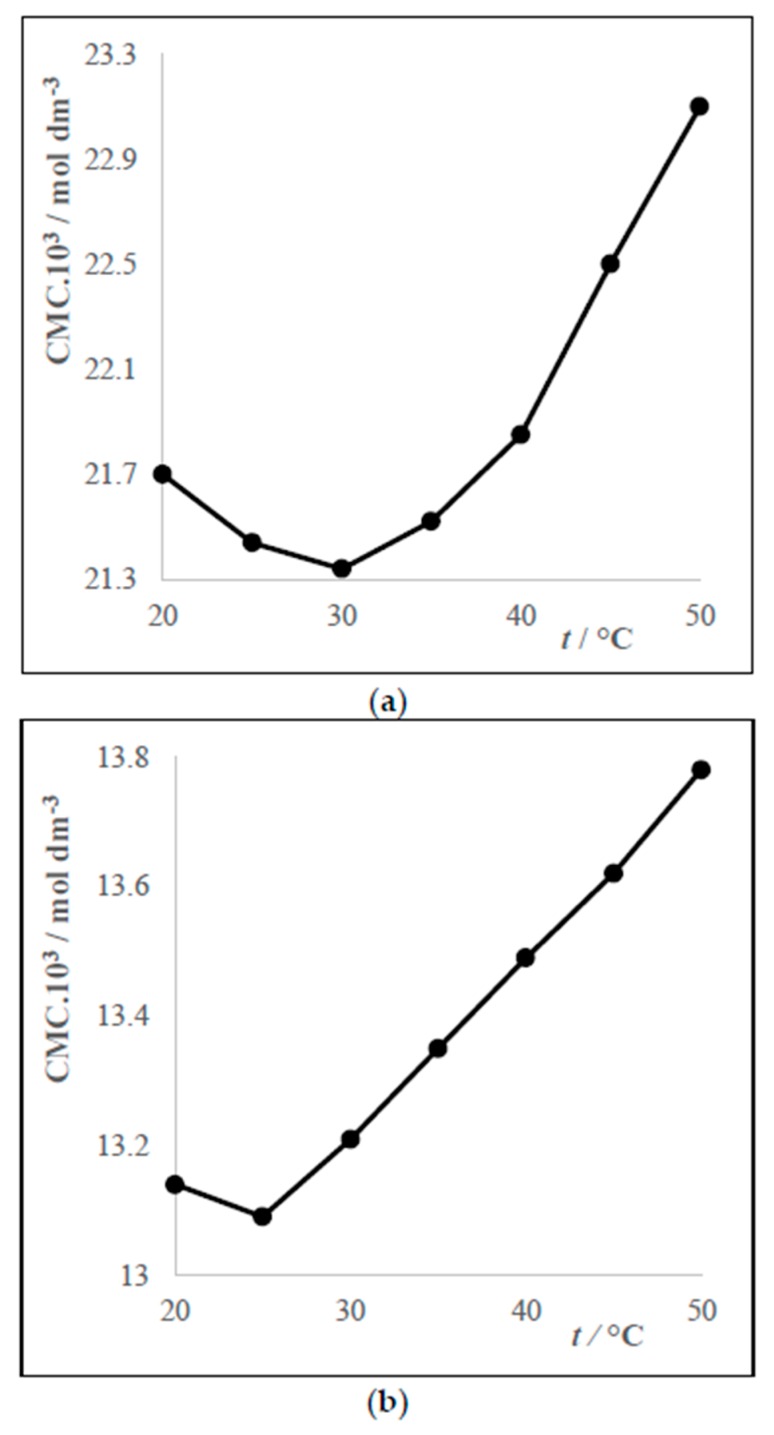
Dependences CMC = *f*(*t*) for systems DDTMACl (II)–water with minimum at 30 °C—part (**a**) and system DDEDMABr (IV)–water with minimum at 25 °C—part (**b**).

It was found that the graphs of the dependences CMC vs. temperature of the studied surfactants have minima at approximately 25 °C or 30 °C. Dependences of the surfactants VI (cis and trans form) are free of minima. Characteristic curves of the dependence CMC = *f*(*t*) are shown in Figure 4.

**Figure 5:**
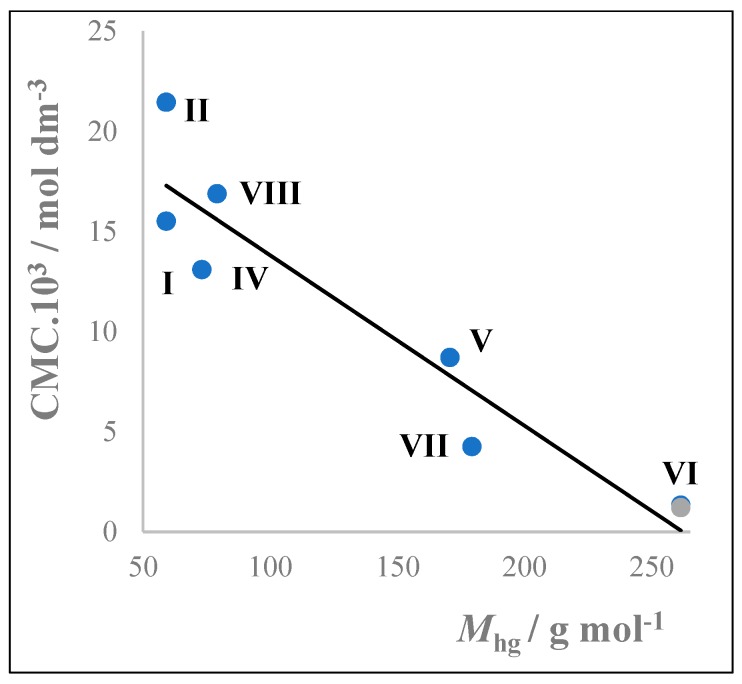
Influences of molar mass of hydrophilic groups (*M*_hg_) on micellization of surfactants at 25 °C. Surfactants III didodecyldimethylammonium chloride which creates vesicles and IX, X are anionic surfactants and are omitted.

The CMCs are different despite the molecule have the same C12 hydrophobic group. The hydrophilic group causes changes of the CMC. This dependence is given in Figure 5.

**Figure 6:**
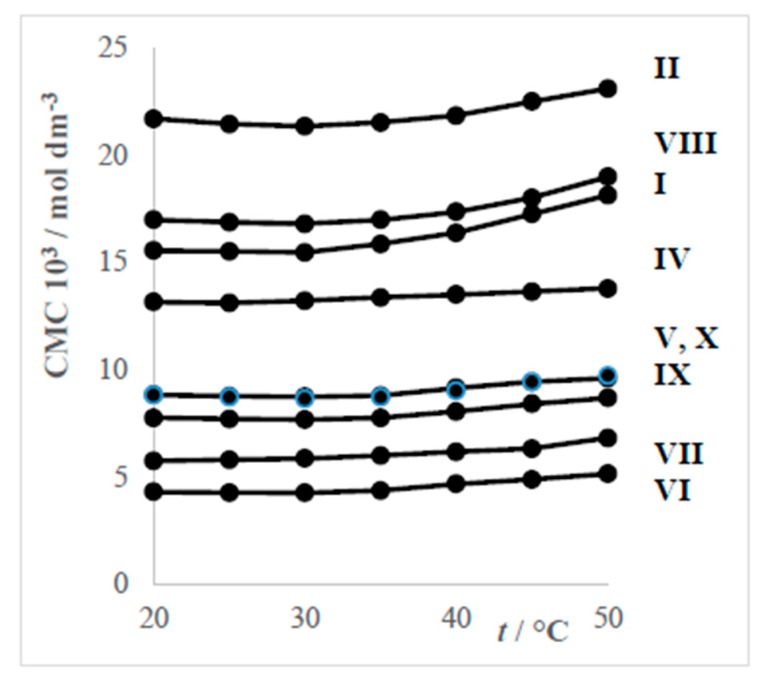
Dependences of CMC on temperature.

Cationic surfactants have higher CMC at the same length of the alkyl chain as anionic ones (Figure 5). Surfactants with chloride anions have higher CMC than those with bromide anions. The CMCs of the cis form of the surfactant VI are higher than they are in the case of the trans form. The Figure 6 shows dependences of CMCs on the temperature.

From Figure 5 and Figure 6 can be deduced that the higher the molecular mass (and probably also volumes) of hydrophilic groups are, the lower the values of CMC reached. The surfactant III is not included in Figure 6, because it has established CVC instead of CMC as shown in Figure 3.

As to the majority of the studied surfactants (except for VI) are commercially available, it may be interesting to compare the CMCs with their counterparts established by some other authors (Table 4).

Let us note that substances VI are not included in the Table 4. That is because substances VI cis and trans were synthetized at the Faculty of Pharmacy in Bratislava. Hence, they are not available for other researchers and for the same reason CMSs cannot be compared with their counterparts obtained elsewhere. As follows from the Table 4, except for a few cases there is a good agreement between critical micellar concentrations (CMC) obtained here and those obtained by other methods and/or other authors. This indicates that the conductometry seems to be a very suitable method.

### 3.2. Determination of Degree of Counterion Binding (β)

From the conductivity curves obtained at various temperatures were calculated not only CMCs but also degrees of ionization (*α*) and, respectively, the degree of counterion binding (*β*). The degrees of ionization are calculated as the ratio of slopes post (*S*_2_) and pre (*S*_1_) of the conductometric curves [15,62].

(8) $$\alpha =\frac{S_{2}}{S_{1}}\;\;\;\;\;\;\beta =1-\alpha$$

The unit of slopes is S m^2^ mol^−1^ while *α* and *β* are unit less quantities. The calculated degrees of counterion binding are shown in Table 5.

In accordance with [15] for ionic surfactants, the increasing temperature accelerates motion of particles in the systems. Values of *β* agree with this observation for all studied surfactant systems.

### 3.3. Determination of Thermodynamic Parameters of Micellization

The thermodynamic parameters of micellization may be calculated in accordance with two models: the model of mass action and the model of phase separation.

Standard molar Gibbs energy of micellization (Δ_m_*G*^0^).

The Δ_m_*G*^0^ may determined by one of the following equations

(9) $$\Delta_{m}G^{0}=RT\ln\mathrm{CMC}$$

(10) $$\Delta_{m}G^{0}=RT\ln x_{\mathrm{CMC}}$$

(11) $$\Delta_{m}G^{0}=\left(1+\beta \right)\;RT\ln\mathrm{CMC}$$

(12) $$\Delta_{m}G^{0}=\left(1+\beta \right)\;RT{\mathrm{lnx}}_{\mathrm{CMC}}$$

(13) $$x_{CMC}=\frac{n_{surf}}{n_{surf}+n_{water}}$$

The symbols have the following meanings: *R*—molar gas constant, T—thermodynamic temperature [K], *x_CMC_*—molar fraction of surfactant in the solution at CMC, Δ_m_*G*^0^—standard molar Gibbs energy of micellization [J mol^−1^], *n*_surf_—number of moles of the surfactant at the concentration equal to CMC [mol], *n*_water_—number of moles of water [mol].

**Figure 7:**
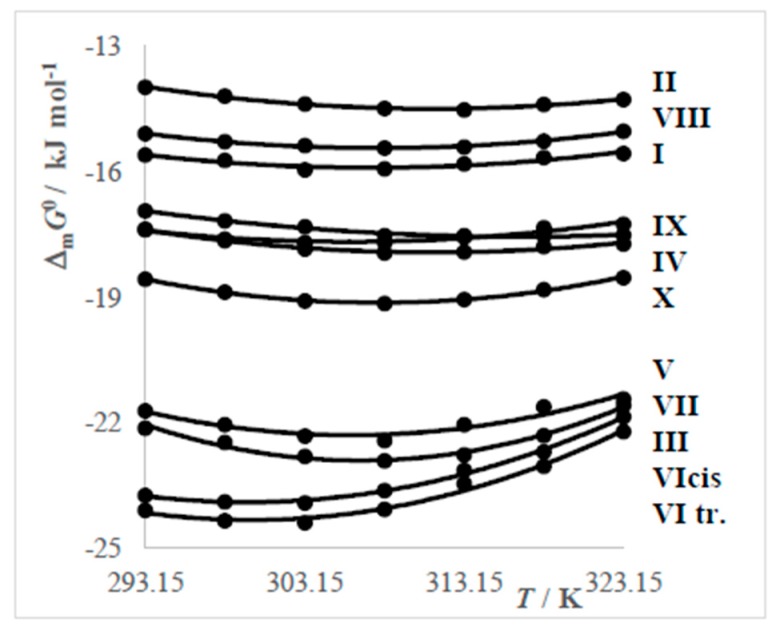
Dependences of Δ_m_*G*^0^ = *f*(*T*) of surfactants in solution.

Because values of the degree of counterion binding have been already established, the standard molar Gibbs energy of micellization can by calculated in accordance to the phase separation model, in accordance with Equation (11). The calculated values of Δ_m_*G*^0^ are in Table 6 and Figure 7.

Values of Δ_m_*G*^0^ (Figure 7) change non-linearly with increasing temperature. All curves reach shallow minima at temperatures 303.15 K or 308.15 K and the changes of Δ_m_*G*^0^ are in the analysed interval very small. Non-linear character of the dependence may be caused by the trend of CMC and β for increasing system temperature. All calculated values of Δ_m_*G*^0^ are negative, indicating a spontaneous process of the micelles’ formation.

Standard molar enthalpy of micellization (Δ_m_*H*^0^).

This thermodynamic parameter is given by the Equation (13).

(14) $$\Delta_{m}H^{0}=\left[\frac{\partial \left(\frac{\Delta_{m}G^{0}}{T}\right)}{\partial \left(\frac{1}{T}\right)}\right]$$

The function ln CMC in the Equation (11) can be approximated by the second-order polynomial equation:

ln CMC = A + B*T* + C*T*^2^ (15)

A, B and C are parameters and CMC is given in units of [mol dm^−3^].

The parameter *β* in Equation (11) depends also on the temperature. Hang at al. [15] suggested to calculate the dependence *β* = *f*(*T*) by Equation (16):

*β* = a + *bT* (16)

where *a* and *b* are parameters. By combining Equations (11) and (14–16) the Δ_m_*H*^0^ may be calculated as follows [23]

(17) $$\begin{array}{cc} \Delta_{m}H^{0} & =-RT^{2}\left[\left(1+\beta \right)\frac{\partial \;lnCMC}{\partial T}+\frac{\partial \beta }{\partial T}\;lnCMC\right]\\ & =-RT^{2}\left[\left(1+\beta \right)\left(B+2CT)+b\;lnCMC\right)\right] \end{array}$$

After inserting Equations (14) and (15) into Equation (16) one can obtain the values of B, C and b. The corresponding coefficients of correlation are in Table 7.

As can be seen from Table 8, all values of Δ_m_*H*^0^ in the analysed temperature interval are negative, which indicates an exothermic process. This means that the major attractive force of micelization of surfactants is hydrophobic interaction.

Contrary to values Δ_m_*G*^0^ which vary (in the range 293.15 K–323.15 K) up to 2 kJ mol^−1^, the changes of Δ_m_*H*^0^ are much more significant and their dependences are linear.

Values of Δ_m_*S*^0^ are positive at low temperatures but at higher temperatures they change from positive to negative. The positive values can be explained [12] by the conveyance of the hydrated hydrophobic part from water to the non-polar core of the micelle and from intensification of the degree of freedom of the hydrophobic part in the associate core. The negative values of Δ_m_*S*^0^ are due to the formation of an iceberg structure nearby CMC.

**Figure 8:**
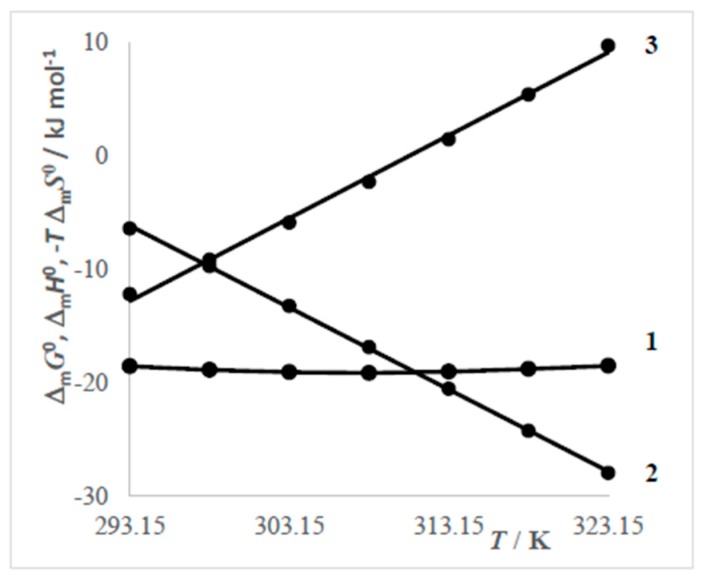
Dependences thermodynamic values on temperature of surfactant V-DDBDMACl: 1—Δ_m_*G*^0^, 2—Δ_m_*H*^0^ a 3—(−*T* Δ_m_*S*^0^).

Dependences of the calculated thermodynamic values of micellization (Δ_m_*G*^0^, Δ_m_*H*^0^ a *T*Δ_m_*S*^0^) on the temperature for the surfactant V-DDBDMAC are shown in Figure 8.

By analysis of Figure 8 one can observe that up to 298.15 K a more significant contribution to Δ_m_*G*^0^ exhibits Δ_m_*H*^0^ and above this temperature it is Δ_m_*S*^0^.

## 4. Conclusions

From the effect of the both hydrophilic groups and temperature on micellar properties and the thermodynamics of micellization of surfactants with 12 carbon chain (dodecyl)—hydrophobic part, the following can be concluded:

Critical micelle concentration:

- Values of CMC depend on composition of the hydrophilic part of surfactants. The higher molar mass of hydrophilic group significantly influences the process of micellization.
- Depending on the packing parameter, the surfactants with a single-long hydrocarbon chain create spherical micelles while those having double-long chains create vesicles.
- Values of CMC depend linearly on molar weights of the hydrophilic group.
- In general, a good agreement exists between values of CMC obtained here and those obtained by other researches and/or different physicochemical methods. This indicates that the conductometry seems to be a very suitable method especially for ionic surfactants.

Degree of counterion binding:

- Calculated values of the counterion binding anions (*β*) decrease linearly with increasing system temperature.
- Thermodynamic values of micellization
- For calculation of thermodynamic values of micellization the pseudo phase model was used.
- Values of Δ_m_*G*^0^ change non-linearly with increasing temperature. All curves reach shallow minima at the temperature 303.15 K or 308.15 K. Changes of Δ_m_*G*^0^ are, within the analysed interval, very small. All calculated values of Δ_m_*G*^0^ are negative what indicates a spontaneous micelle formation.
- All values of Δ_m_*H*^0^ are, within the analysed temperature interval, negative, which indicates an exothermic process. It means that a major attractive force of micellization of surfactants comes from the hydrophobic interaction.
- Values of Δ_m_*S*^0^ are positive at low temperatures but at higher temperatures they change from positive to negative.
- By analysing of the experiments one can observe that at the temperatures below 298.15 K a more significant contribution to Δ_m_*G*^0^ is caused by Δ_m_*H*^0^ and above this temperature it is Δ_m_*S*^0^.

## Figures and Tables

**Table 1:** Fundamental characteristic of studied compounds.

Surfactant	Name of Substance	Supplier	*M*_c_ (g mol^−1^)	*M*_hg_ (g mol^−1^)	m.p. (°C)
DDTMABr I	Dodecyltrimethylammonium bromide	Aldrich	308.35	59.12	246
DDTMACl II	Dodecyltrimethylammonium chloride	Fluka Chemika	263.90	59.12	246
DDDMABr III	Didodecyldimethylammonium bromide	Fluka Chemika	462.65	44.09	157–162
DDEDMABr IV	Dodecylethyldimethylammonium bromide	Fluka Chemika	350.43	73.14	149–151
DDBDMACl V	Dodecylbenzyldimethylammonium chloride	Fluka Chemika	384.45	170.66	60
DBDMABr cis, trans VI	Dodecyl-[(2-benzyloxy)-cyclohexylmethyl]-N,N-dimethylammonium bromide	*	497.58	261.37	127–129
DDDMEBr VII	Dodecylmethylephedrinium bromide	Fluka Chemika	428.50	179.26	104–106
DDPCl VIII	Dodecylpyridinium chloride	Fluka Chemika	284.00	79.10	66–70
DSNa IX	Sodium dodecyl sulphate	Merck	288.38	96.06	206
DSLi X	Lithium dodecyl sulphate	Merck	272.32	96.06	859

* Compounds were synthetized at the Faculty of Pharmacy, Comenius University in Bratislava

**Table 2:** Calculated values of critical micellar concentrations (CMC) of all studied surfactants at temperature 20–50 °C.

(CMC ± s_CMC_ ) 10^3^ [ mol dm^−3^]							
Surfactant							
t [°C]	20	25	30	35	40	45	50
I	15.54 ± 0.02	15.50 ± 0.02	15.45 ± 0.02	15.85 ± 0.01	16.38 ± 0.04	17.25 ± 0.02	18.12 ± 0.03
II	21.70 ± 0.03	21.44 ± 0.04	21.34 ± 0.02	21.52 ± 0.04	21.85 ± 0.03	22.50 ± 0.04	23.10 ± 0.05
III CVC_1_ * CVC_2_ CVC_3_	7.29 ± 0.02	7.26 ± 0.03 1.66 ± 0.05 0.078 ± 0.04	7.21 ± 0.04	7.29 ± 0.02	7.52 ± 0.04	8.30 ± 0.03	9.17 ± 0.03
IV	13.14 ± 0.04	13.09 ± 0.03	13.21 ± 0.03	13.35 ± 0.03	13.49 ± 0.04	13.62 ± 0.05	13.78 ± 0.03
V	8.80 ± 0.03	8.70 ± 0.02	8.62 ± 0.02	8.69 ± 0.03	9.00 ± 0.05	9.42 ± 0.02	9.72 ± 0.04
VI cis	1.27 ± 0.03	1.32 ± 0.03	1.39 ± 0.02	1.54 ± 0.03	1.81 ± 0.03	2.04 ± 0.04	2.54 ± 0.03
trans	1.17 ± 0.02	1.20 ± 0.03	1.26 ± 0.03	1.42 ± 0.03	1.70 ± 0.03	1.93 ± 0.03	2.41 ± 0.04
VII	4.28 ± 0.03	4.25 ± 0.03	4.23 ± 0.04	4.35 ± 0.03	4.65 ± 0.03	4.86 ± 0.04	5.12 ± 0.05
VIII	16.98 ± 0.02	16.87 ± 0.02	16.80 ± 0.05	16.98 ± 0.04	17.35 ± 0,05	18.00 ± 0.03	18.98 ± 0.04
IX	7.73 ± 0.03	7.68 ± 0.02	7.65 ± 0.03	7.74 ± 0.03	8.02 ± 0.04	8.39 ± 0.05	8.66 ± 0.04
X	8.81 ± 0.03	8.74 ± 0.03	8.71 ± 0.02	8.78 ± 0.03	9.13 ± 0.04	9.41 ± 0.03	9.58 ± 0.03

s_CMC_—the standard deviation was calculated for three individual measurements. * critical vesicle concentration.

**Table 3:** Calculated values of *l_C_*, *V_0_*_,_ *a_0_, r, N_agg_* and *p.*

Alkyl	*l*_C_ [Å]	*V*_0_ [Å^3^]	*N_Agg_*	*r* [Å]	*a*_0_ [Å^2^]	*p*
Dodecyl	16.68	350.2	55.50	16.60	63.30	0.33
Didodecyl	16.68	703.5	27.63	34.07	61.94	0.68

**Table 4:** Comparison of established CMCs with those obtained by some other authors.

CMC 10^3^ [mol dm^−3^]									
Surfactant									
t [°C]	20	25	30	35	40	45	50	Met.	Lit.
I	15.54	15.50	15.45	15.85	16.38	17.25	18.12	κ	present res.
			15.00					κ	[18]
			11.40					γ	
	15.20	15.50	15.90	16.20	16.50			γ	[19]
	15.00	15.30	15.70	15.90	16.20			γ	[20]
	10.07							κ	[21]
	13.49							κ	[22]
		14.50						κ	[23]
		14.60						γ	
		15.80		16.40		17.00		κ	[24]
		15.90						f	
					15.70			κ	[25]
		10.70						NMR	[26]
		15.00						ss	[27]
		15.60						κ	[10]
II	21.70	21.44	21.34	21.52	21.85	22.50	23.10	κ	present res.
			21.20					κ	[28]
			21.40					γ	
	22.98	22.6	21.51	21.80	23.33	23.81		H	[29]
	20.12	19.35	18.53	16.65	19.76	19.78		κ	
	22.60	22.20	21.70	21.90	22.20	22.70		κ	[30]
		21.00						κ	[31]
		21.00						κ	[32]
	22.10	21.30	20.40	19.60				κ	[33]
III CVC_1_ CVC_2_ CVC_3_ CVC_3_ CVC_2_ CVC_3_ CVC_3_	7.29	7.26 1.66 0.079 0.050 0.70 0.048 0.050	7.21	7.29	7.52	8.30	9.17	κ turb.	[63] [64] [65]
IV	13.14	13.09	13.21	13.35	13.49	13.62	13.78	κ	
		14.00						κ	
		14.40						κ	[34]
		9.50	10.50	11.00	13.50	14.00	17.00	*ρ*	[35]
		9.30	10.00	10.80	11.30	12.00	15.30	η	
		13.00	14.00	15.00	15.55	15.80	16.50	uv	
		13.00	14.30	15.00	15.50	16.00	17.00	κ	
V	8.80		8.62	8.69	9.00	9.42	9.72		present res.
	8.80	8.83	8.99						[36]
		8.70							[37]
VII	4.28	4.25	4.23	4.35	4.65	4.86	5.12	κ	present res.
		3.94	4.04	4.16	4.34	4.54	5.00	κ	[38]
			4.05		4.50		4.99	EMV	
			4.10		4.60		5.02	UV	
			4.12		4.40		5.00	γKrus	
			4.08		4.53		5.00	γdrop	
VIII	16.98	16.87	16.80	16.98	17.35	18.00	18.94	κ	present res.
		16.80	18.00		19.10		20.00	κ	[39]
		16.50						κ	[37]
		16.20						κ	[40]
		17.13						γ	[41]
		17.68						κ	
	19.20	17.20	13.70	19.80	21.70			κ	[42]
	18.90	18.00	18.50		20.00		21.00	κ	[43]
	16.70	15.60	17.50		18.20		19.60	γ	
		16.20	17.29	17.80	18.41	19.42		κ	[44]
		15.00						κ	50
			16.00					κ	51
IX	7.73	7.68	7.65	7.74	8.02	8.39	8.66	κ	present res.
			8.30					κ	[37]
		9.16	7.96	4.49	3.70			γ	[47]
		7.98						κ	[48]
		7.96						κ	[49]
		7.80		8.60		9.80	10.60	κ	[50]
	8.70	8.20	8.65	8.90	9.00	9.10		κ	[51]
		8.10						f	
		8.11						UV	
		8.50						UV	[52]
		8.26						κ	[53]
		8.26						f	
		8.25						γ	[54]
		8.85						κ	
		8.85						f	
	8.70	8.20	8.65	8.90	9.00	9.10		κ	[55]
		8.30			7.70		8.10	H	[56]
		8.10			8.70		9.20	γ	
X	8.81	8.74	8.71	8.78	9.13	9.41	9.58	κ	present res.
		8.98						κ	
		7.12						γ	[57]
	9.09	8.98	9.06	9.21	9.32	9.39		κ	[58]
		8.96		9.28				ρ	
		8.93		9.18				c	
			8.77					κ	[59]
		8.90						κ	[60]

κ—conductivity, γ—surface tension, ss—speed of sound, *ρ*—density, η—viscosity, uv—ultrasound velocity, EMV—electromotorical voltage, UV—ultraviolet light, f—fluorimetry, H—enthalpy, c—adiabatic compressibility, turb—turbidity.

**Table 5:** Calculated values of degrees of counterion binding for all surfactant solutions at the temperature 20–50 °C.

β							
Surfactant							
t [°C]	20	25	30	35	40	45	50
I	0.536	0.522	0.518	0.496	0.477	0458	0.444
II	0.497	0.490	0.482	0.473	0.458	0.433	0.409
III	0.752	0.748	0.742	0.730	0.709	0.689	0.650
IV	0.604	0.597	0.587	0.584	0.563	0.542	0.521
V	0.609	0.605	0.593	0.574	0.553	0.524	0.488
VI cis	0.460	0.453	0.442	0.423	0.407	0.385	0.361
trans	0.464	0.460	0.449	0.432	0.412	0.393	0.371
VII	0.633	0.628	0.620	0.609	0.576	0.534	0.512
VIII	0.519	0.510	0.492	0.478	0.459	0.436	0.411
IX	0.465	0.459	0.440	0.420	0.397	0.371	0.352
X	0.507	0.501	0.492	0.478	0.465	0.441	0.419

Values of *β* decrease with decreasing value of the molecular mass of the hydrophilic group.

**Table 6:** Calculated values of standard molar Gibbs energy of micellization of all surfactants.

Δ_m_*G*^0^ [kJ mol^−1^]							
Surfactant							
T [K]	293.15	298.15	303.15	308.15	313.15	318.15	323.15
I	−15.59	−15.70	−15.95	−15.92	−15.81	−15.66	−15.56
II	−13.97	−14.19	−14.37	−14.49	−14.51	−14.38	−14.26
III	−22.12	−22.47	−22.80	−22.90	−22.76	−22.30	−21.57
IV	−16.93	−17.16	−17.31	−17.52	−17.52	−17.52	−17.51
V	−18.56	−18.88	−19.09	−19.14	−19.05	−18.80	−18.22
VI cis	−23.73	−23.88	−23.91	−23.61	−23.13	−22.69	−21.85
trans	−24.09	−24.34	−24.38	−24.06	−23.44	−23.03	−22.20
VII	−21.71	−22.04	−22.32	−22.41	−22.04	−21.61	−21.43
VIII	−15.09	−15.28	−15.37	−15.43	−15.40	−15.26	−15.03
IX	−17.36	−17.61	−17.69	−17.68	−17.55	−17.34	−17.25
X	−17.38	−17.63	−17.84	−17.93	−17.91	−17.78	−17.77

**Table 7:** Calculated parameters of dependence ln CMC = f(*T*) – B and C (Equation 15), *β* = f(*T*) – b (Equation 16) and correlation coefficients.

Equation	(15)			(16)		
Parameter	B	C	R *	b	S_b_ **	r
I	−0.15573	0.00026	0.998	−0.00318	0.00019	0.990
II	−0.10799	0.00018	0.997	−0.00287	0.00034	0.970
III	−0.38990	0.00065	0.996	−0.00326	0.00049	0.946
IV	−0.02161	0.00004	0.991	−0,00273	0,00032	0.970
V	−0.15551	0.00026	0.989	−0.00293	0.00029	0.977
VI cis	−0.37643	0,00065	0.998	−0.00384	0.00025	0.986
trans	−0.41442	0.00071	0.998	−0.00375	0.00021	0.992
VII	−0.17483	0.00029	0.989	−0.00250	0.00060	0.990
VIII	−0.15025	0.00025	0.998	−0.00361	0.00022	0.992
IX	−0.13788	0.00023	0.992	−0.00399	0.00023	0.991
X	−0.10241	0.00017	0.977	−0.00293	0.00021	0.987

r *—coefficient of correlation, s_b_ **—standard deviation of the slope b, Equation (16). The standard molar enthalpy of micellization is shown in Table 8.

**Table 8:** Calculated values of standard molar enthalpy of micellization for all surfactant. solutions at temperature interval 20–50 °C.

Δ_m_*H*^0^ [kJ mol^−1^]							
Surfactant							
**T [K]**	293.15	298.15	303.15	308.15	313.15	318.15	323.15
**I**	−5.85	−9.14	−12.34	−15.73	−19.22	−22.78	−26.50
**II**	−5.23	−7.43	−9.73	−12.12	−14.55	−17.06	−19.59
**III**	−1.04	−9.51	−18.52	−27.90	−37.56	−47.37	−57.03
**IV**	−10.56	-11.39	−12.23	−13.11	−13.97	−14.85	−15.76
**V**	−6.38	−9.71	−13.23	−16.85	−20.53	−24.23	−27.97
**VI cis**	−23.16	−30.80	−38.76	−46.78	−54.94	−63.33	−75.52
**trans**	−20.02	−28.30	−36.90	-45.59	−54.32	−62.51	−72.54
**VII**	−4.14	−7.80	−11.67	−15.68	−19.68	−23.72	−27.98
**VIII**	−6.52	−9.58	−12.78	−16.08	−19.45	−22.87	−26.30
**IX**	−10.69	−13.57	−16.58	−19.65	−22.72	−25.82	−29.09
**X**	−6.95	−9.11	−11.37	−13.71	−16.07	−18.49	−21.01
